# Application of the Analytical Procedure Lifecycle Concept to a Quantitative ^1^H NMR Method for Total Dammarane-Type Saponins

**DOI:** 10.3390/ph16070947

**Published:** 2023-06-29

**Authors:** Wenzhu Li, Jiayu Yang, Fang Zhao, Xinyuan Xie, Jianyang Pan, Haibin Qu

**Affiliations:** 1 Pharmaceutical Informatics Institute, College of Pharmaceutical Sciences, Zhejiang University, Hangzhou 310058, China; wenzhu_li@zju.edu.cn (W.L.); yangjy98@zju.edu.cn (J.Y.); z_fang@zju.edu.cn (F.Z.); xiexy96@zju.edu.cn (X.X.); panjy@zju.edu.cn (J.P.); 2 State Key Laboratory of Component-Based Chinese Medicine, Innovation Center, Zhejiang University, Hangzhou 310058, China

**Keywords:** analytical procedure lifecycle, AQbD, ^1^H NMR, quantitative NMR, dammarane-type saponins

## Abstract

Dammarane-type saponins (DTSs) exist in various medicinal plants, which are a class of active ingredients with effects on improving myocardial ischemia and immunomodulation. In this study, a quantitative ^1^H NMR method of total DTSs in herbal medicines was developed based on the analytical procedure lifecycle. In the first stage (analytical procedure design), the Ishikawa diagram and failure mode effects and criticality analysis were used to conduct risk identification and risk ranking. Plackett–Burman design and central composite design were used to screen and optimize critical analytical procedure parameter. Then, the method operable design region was obtained through modeling. In the second stage (analytical procedure performance qualification), the performance of methodological indexes was investigated based on analytical quality by design. As examples of continued procedure performance verification, the method was successfully applied to determine the total DTSs in herbal pharmaceutical preparations and botanical extracts. As a general analytical method to quantify total DTSs in medicinal plants or pharmaceutical preparations, the developed method provides a new quality control strategy for various products containing dammarane-type saponin.

## 1. Introduction

Dammarane-type saponins (DTSs), classified as tetracyclic triterpene saponins, are widely distributed in Araliaceae and Cucurbitaceae herbs, such as *Ginseng*, *American Ginseng*, *Panax notoginseng*, and *Gynostemma*. Thus far, more than 760 DTSs have been reported from more than 130 plant species [1]. Studies have revealed that DTSs possess a diverse range of effects including antitumor, anti-inflammatory, myocardial ischemic, immunomodulatory, hypoglycemic, anti-shock, hepatoprotective, sedative, and tranquilizing properties [2], so DTSs are considered as crucial active pharmaceutical ingredients (APIs) in herbal preparations. In the standards of relevant preparations, the content of total saponins is specified as a critical quality evaluation indicator. The glacial acetic acid–perchloric acid–UV chromogenic method is a classical approach for determining total saponins content. However, this method is laborious, necessitates the use of highly corrosive reagents, and can only provide fluctuating estimated outcomes, rather than absolute molar concentrations [3]. Several studies have reported the simultaneous determination of up to 10 different DTSs using HPLC or UPLC methods [4,5,6]. Nonetheless, it has been shown that some preparations contain more than 50 kinds of DTSs [7]. Therefore, it is imperative to make further advancements in methods development for the analysis of DTSs that are more efficient, accurate, and comprehensive.

Li et al. [3] developed a proton quantitative nuclear magnetic resonance (^1^H qNMR) method for the determination of total ginsenosides. Although this method has limitations in its scope, as it cannot be directly applied to other classes of DTSs or other herbal species, it still demonstrates the potential of qNMR methods for the analysis of DTSs. ^1^H qNMR spectroscopy is a non-destructive and rapid analytical technique with quantitative ability of high accuracy and good reproducibility. Due to the broad application prospect of qNMR in the analysis of complex systems, this study aims to develop a ^1^H qNMR method for the determination of DTSs in herbal medicines. In recent years, several standards and guidelines have recommended the application of lifecycle management to the analytical methodology development procedure in order to obtain a more robust parameter space [8,9].

The concept of lifecycle management was mentioned in 1975 for the first time in the scientific literature [10] and has been introduced into the field of chemical analysis in the 21st century [11]. In 2021, the United States Pharmacopeia (USP-2021) included the General Chapter <1220> “*The Analytical Procedure Lifecycle (APLC)*” formally, which addresses the application of lifecycle management to the analytical procedure [8]. APLC integrates and extends the steps of analytical method development, validation, verification, transfer, and maintenance in traditional procedures, which are regarded as a continuous dynamic cycle to ensure valid data generation throughout its lifecycle. Analytical target profile (ATP) is a fundamental component of the lifecycle approach to analytical procedures, which determines the criteria for the application purpose and the expected performance characteristics of the analytical measurements [8]. There are three stages of the lifecycle approach to analytical procedures, namely analytical procedure design (APD), procedure performance qualification (APPQ), and continued procedure performance verification (CPPV). In the first stage (APD), quality risk management (QRM) means are applied to carry out risk analysis and risk ranking of parameters. Then, the design of experiments (DoE) methods are applied to optimize critical analytical procedure parameters (CAPPs) and obtain the method operable design region (MODR) based on the concept of analytical quality-by-design (AQbD). In the second stage (APPQ), verification experiments were conducted to confirm that the method will operate (in routine use) as intended and meets the previously defined ATP criteria [11]. In the last stage (CPPV), the established method is continuously monitored and validated in its daily application based on a set of analytical control strategies.

The attempts to apply APLC In pharmaceutical analysis-related studies have gradually increased in the past decade, but reports involving the analysis of herbal medicines are still relatively few [12]. Most of the analytical methods for herbal medicines developed by applying APLC and AQbD are chromatographic methods aiming at the separation and quantification of active ingredients of herbal medicines. For example, there are studies about the development of HPLC-UV/PDA methods for the analysis of flavonoids [13,14], saponins [15,16], terpenoids [17], quinones [9], and phenolic [18]; HPLC-MS methods to determine terpene lactones [19] and polyphenols [20]; HPLC-ELSD methods to quantify sugars and their derivatives [21], etc. In addition, other chromatographic methods have also been developed based on AQbD, such as capillary electrophoresis [22,23], supercritical fluid chromatography [24,25], gas chromatography [26,27], etc. However, the practice of applying APLC and AQbD to developing qNMR methods for herbal medicines has not been reported.

In this study, APLC and AQbD approaches were applied to develop a quantitative ^1^H NMR (^1^H qNMR) method for total DTSs in herbal medicines. Failure mode effects and criticality analysis (FMECA) was used for risk assessment. Plackett–Burman design (PBD) [28] and central composite design (CCD) were applied to screen and optimize CAPPs. The developed method has been successfully applied to quantify total DTSs in a liquid preparation, a solid preparation, and a production process intermediate, and has performed well in the validation of all the methodological indexes.

## 2. Results and Discussion

### 2.1. Risk Identification and Assessment Results Based on the Ishikawa Diagram and FMECA

**Figure 1:**
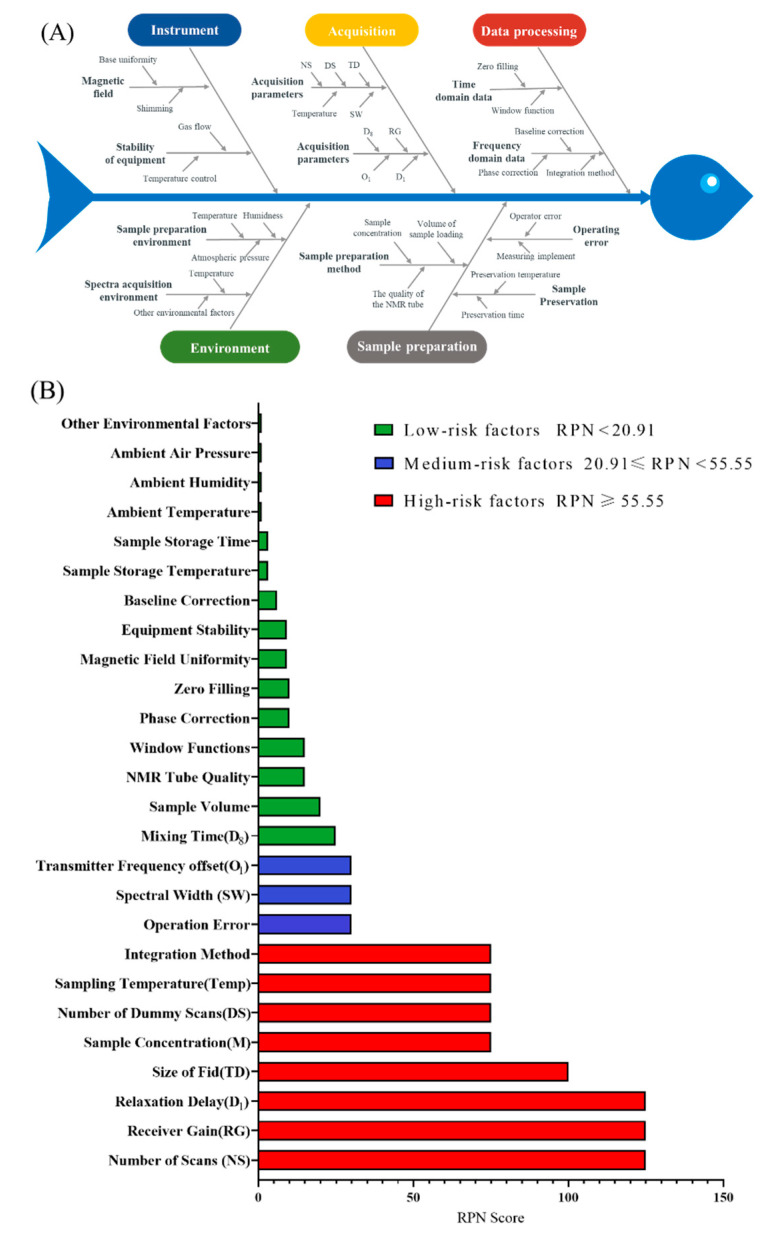
(**A**) Risk identification for the ^1^H qNMR method based on Ishikawa diagram; (**B**) Risk ranking of the ^1^H qNMR method based on FMECA.

In this study, risk factors that may affect the results of the ^1^H qNMR method were analyzed as potential failure factors of FMECA from five aspects, including sample preparation, spectra acquisition, data processing, environment, and instrument, which were summarized in an Ishikawa diagram (Figure 1A) [12]. Risk factors identified in the Ishikawa diagram were evaluated by FMECA. The detailed process of FMECA was shown in Section S4. The definition of failure mode for the ^1^H qNMR method as well as S, O, D, and RPN scores of each factor were listed in Tables S2 and S3. As shown in Figure 1B, the maximum value of RPN was 125, the minimum value is 1, and the standard deviation (SD) was 42.88. Considering the 95% confidence interval, the statistical mean of RPN was 20.91~55.55. Based on the RPN score, risk factors could be divided into three levels: low, medium, and high. The high-risk factors for the method should be investigated and optimized in the method development process.

From the RPN calculation results, it was clear that sample concentration (M), number of dummy scans (DS), number of scans (NS), sampling temperature (Temp), size of fid (TD), receiver gain (RG), relaxation delay (D_1_), and integration method were the high-risk factors that need to be further investigated and strictly controlled. Among these factors, only the integration method was a data processing parameter, while the others were experimental parameters. Since there was no interaction between experimental and data processing parameters, the integration method would be examined separately from the other high-risk factors. Based on literature research [29] and single-factor experimental comparisons, the “line fitting” method in the MestReNova software was selected as the integration method. The remaining seven parameters will be further screened and optimized by DoE as potential CAPPs.

### 2.2. DoE-Based Analytical Process Design (APD) 

#### 2.2.1. CAPPs Screening Results Based on PBD

##### PBD Screening Experiments

The total of 15 runs for screening experiments were obtained by PBD, which were used to investigate the effects of M (*x*_1_), DS (*x*_2_), NS (*x*_3_), TD (*x*_4_), RG (*x*_5_), D_1_ (*x*_6_), and Temp (*x*_7_) on the ^1^H qNMR method. Considering that the most important expected requirements in the ATPs are good accuracy and precision, and precision is mainly related to signal resolution and signal intensity, accuracy, SNR, and resolution were used as evaluation indexes of CAPPs (*y*). Parameters of screening experiments in PBD and the coding values of each factor were shown in Table 1.

The samples used for PBD experiments were obtained by mixing representative DTSs ginsenoside Rb1, ginsenoside Rg1, ginsenoside Re, notoginsenoside R1, notoginsenoside Fe, gypenoside XLIX, gypenoside XVII, and gypenoside XLVI. The above mixing DTSs were dissolved in CD_3_OD containing 1.46 mmol/L DMT to prepare samples with total DTSs concentrations of 5, 2.5, and 0.5 mmol/L, respectively. The ^1^H NMR spectra of all samples were recorded with a Bruker AVANCE III 500 MHz NMR spectrometer (Bruker Technologies GmbH, Germany, 5 mm BBO probe with Topspin workstation). A classical water suppression pulse ZGPR was used to optimize the parameter of transmitter frequency offset (O_1_) at different temperatures, and the optimization results were 2436.22, 2428.67, and 2416.84 Hz at 296, 298, and 300 K, respectively. Then, NOESYGPPR1D pulse was used to acquire ^1^H NMR spectra, and the parameters were set according to the experimental conditions in Table 1. The parameters not specified in the table were set as follows: pulse width (SW) was 12.016 ppm, mixing time (D_8_) was 0.05 s, and the 90° pulse width (P_1_) was 14.75 μs. Finally, a total of fifteen ^1^H NMR spectra were acquired, and the data were processed according to the method in Section 3.2.5.

##### CAPPs Screening Results

The absolute value of the percentage of relative deviation between the measured value of ^1^H qNMR and the actual weighing value of the DTSs content in the samples was used as the accuracy index. The peak width factor of the quantitative peaks (calculated by Mestrenova software (version 14.0.0)) was used as the resolution index; the ratio of the internal standard peak height to the noise level (calculated by Mestrenova software (version 14.0.0)) was used as the SNR index. The results of the indexes for the 15 PBD experiments are shown in Table 2. The analytical parameters with significant effects on the four indexes were selected as CAPPs by using the weighted multiple linear regression coefficient method. The multiple linear regression model between the analytical parameters (*x_i_*) and the evaluation indexes (*y*) is represented by Equation (1), where $a_{0}$ is a constant term and $x_{i}$ and $a_{i}$ are the level and partial regression coefficients of the corresponding analytical parameters, respectively.

(1) $$y=a_{0}+\overset{7}{\underset{i=1}{\sum }}a_{i}x_{i}$$

**Figure 2:**
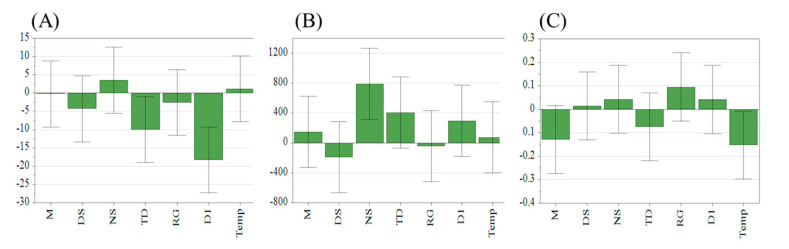
Regression coefficient of multiple linear models of (**A**) Accuracy, (**B**) SNR, and (**C**) resolution.

After fitting, the regression coefficients corresponding to the three evaluation indexes are shown in Figure 2, and the determination coefficients (r^2^) are 0.644, 0.705, and 0.787, respectively. The error line represented the 95% confidence interval for the results of the 15 experiments. If the range of this confidence interval contains 0, the parameter was not significant for the model. As shown in Figure 2, the significant parameter in the SNR model was NS, the significant parameters in the accuracy model were D_1_ and TD, and the significant parameter in the resolution model was Temp. The principles of significant parameters affecting the three evaluation indexes were further analyzed. A ^1^H NMR spectrum was usually superimposed by multiple acquisitions, and a larger NS led to higher signal intensity and SNR. D_1_ affected the time interval between two acquisitions. A too small D_1_ resulted in incomplete relaxation of the signals, which affected accuracy. TD reflected the inherent resolution of the spectra, and a sufficient amount of data points (TD) could accurately describe the shape of the signal peaks. Temperature affected the velocity of molecular motion, thus affecting the characteristic displacement of the molecule. Strengthening the temperature control means could ensure a good signal resolution, so there is no need to further optimize the temperature setting. Finally, the three remaining significant parameters NS, TD, and D_1_ were selected as CAPPs for this study.

#### 2.2.2. The Results of CCD-based CAPP Optimization

##### CCD Experimental Design Results

After determining the CAPPs (NS, TD, and D_1_), this study used CCD experiments to find the combination of parameters that ensure robust operation. Before that, a parameter closely related to TD and D_1_ is considered: acquisition time (AQ). AQ is not a directly set parameter but is determined by SW and TD, as shown in Equation (2):

(2) $$\frac{1}{AQ}=\frac{2SW}{TD}$$

The *time lag* (*T_l_*) between two acquisitions is the sum of AQ and D_1_. D_1_ affects the accuracy by influencing *T_l_*, but the change of TD also affects *T_l_*. In order to avoid the interaction between TD and D_1_ involving the modeling, the three factors in CCD were set as NS (X_1_), TD (X_2_), and *T_l_* (X_3_ = AQ + D_1_). The parameter levels of NS and TD were determined with reference to the literature and previous experimental experience, and the level of *T_l_* was set according to the longest (longitudinal relaxation time) T_1_ of the internal standard signal and quantitative signals. The final design yielded 17 optimized experiments with 3 factors and 5 levels. The CCD experiments table and the coding values of each factor are shown in Table 3.

The evaluation indexes of CCD experiments were determined based on ATPs and the results of PBD experiments. The accuracy and SNR indexes are the same as those of the PBD experiments. Since the NS, TD, and D_1_ were not significant in the resolution model, resolution was no longer used as an evaluation index. Considering the requirement of ATPs on the analysis time, the total acquisition time (T_q_) was included in the evaluation index of the optimization experiment.

The sample used for the CCD experiments was the sample with total DTSs concentrations of 5 mmol/L in Section 2.2.1. The ^1^H NMR spectrum was acquired with NOESYGPPR1D pulse. The acquisition parameters were set according to Table 3, and the parameters not specified in the table were set as follows: SW at 12.016 ppm, Temp at 298 K, O_1_ at 2428.67 Hz, P_1_ at 14.75 μs, DS at 4, and RG at 71.8.

##### MODR Calculation Results Based on the Optimization Model

The results of indexes for the 17 CCD experiments were shown in Table 3. MODDE 13 software (Umetrics Inc., Sartorius AG, Göttingen, Germany.) was used to establish the PLS regression models between the parameters and the evaluation indexes, as shown in Equation (3). Where $b_{0}$ were constants, $b_{i}$, $b_{ii}$, and $b_{ij}$ were regression coefficients, Y was the evaluation index of the CCD optimization experiments, $X_{i}$ and $X_{j}$ were the coded values of each CCD factor. The determination coefficients, r^2^ and adjusted r^2^ (r^2^_adj_), are shown in Table 4. The r^2^ was greater than 0.85 for all three models, which proved that the models fitted well.

(3) $$Y=b_{0}+\overset{3}{\underset{i=1}{\sum }}b_{i}X_{i}+\overset{3}{\underset{i=1}{\sum }}b_{ii}{X_{i}}^{2}+\overset{2}{\underset{i=1}{\sum }}\overset{3}{\underset{i=1}{\sum }}b_{ij}X_{i}X_{j}$$

**Figure 3:**
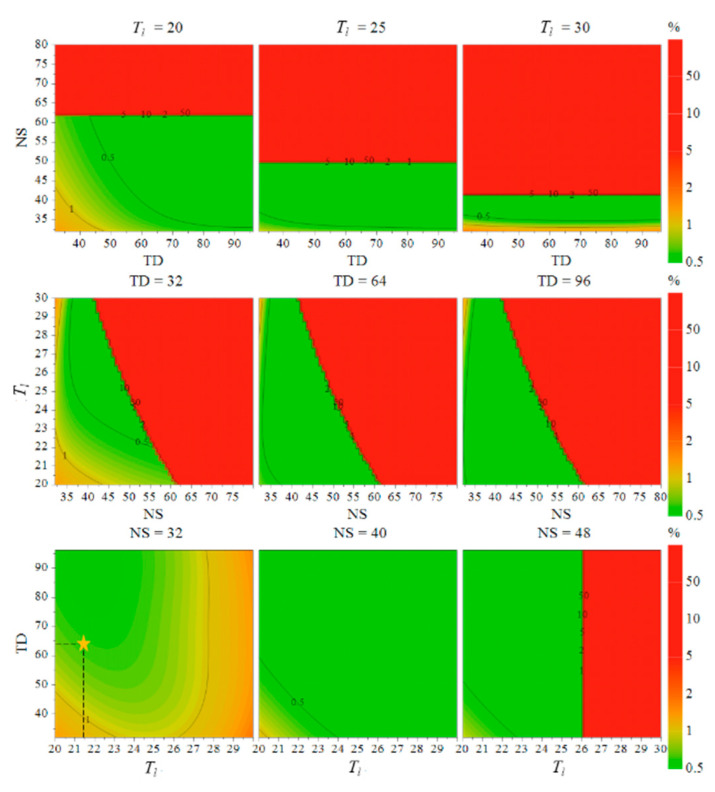
Design space of the total DTSs ^1^H qNMR method.

Based on the above models, the Monte Carlo method was applied to calculate the probability-based design space. According to the ATPs, the upper limit of accuracy was set at 3%, the lower limit of SNR was set at 1000, and the upper limit of analysis time was set at 25 min. The calculation parameters were set as follows: the resolution of the simulation set point was 64, the number of simulations calculated for each point was 10,000 times, and the failure probability was 1%. The calculation results are shown in Figure 3. The gradient of color from green to red in the figure indicated that the probability of failure of each index under the corresponding parameter conditions (i.e., the probability of failure) gradually increases from 0.5%. The contour lines and the numbers on the lines in the figure represented the probability of failure of each evaluation index in the corresponding area. In this study, the region with a probability of failure less than or equal to 1% was used as the design space (the green region in Figure 3), and the region that was convenient for practical operation was selected as MODR. MODR was set as follows: TD was 65,536 or 98,304; NS was 32, 40, or 48; and *T_l_* was 20~25 s. 

### 2.3. Procedure Performance Qualification (APPQ)

#### 2.3.1. Determination of Acquisition Parameters

According to ATPs, the analysis time needed to be as short as possible while ensuring the robustness of the results. Therefore, the two parameters related to the analysis time, NS and *T_l_*, should be as small as possible. The final selection of parameter combination is shown as the star point in Figure 3, and the total duration of acquisition was 13 min. The acquisition parameters were determined as follows: ^1^H NMR spectrum was acquired with a NOESYGPPR1D pulse, with a solvent CD_3_OD locking field, SW of 12.016 ppm, Temp of 298 K, DS of 4, NS of 32, D_1_ of 18.73 s, TD of 65,536, O_1_ of 2428.67 Hz, D_8_ of 0.05 s, P_1_ of 14.75 μs, RG of 14.75 μs, and RG of 0.05 s.

#### 2.3.2. APPQ Index Examination

##### MODR Robustness Examination

To verify the robustness of MODR, six experimental sites were selected in MODR to conduct validation experiments. The CAPPs (NS, TD, D_1_) settings for the six experiments were (32, 98,304, 19 s), (48, 98,304, 16 s), (40, 98,304, 17 s), (48, 65,536, 20 s), (32, 65,536, 18 s), and (40, 65,536, 19 s). The other parameters are set according to Section 2.3.1. The ^1^H NMR spectra were collected with the above parameters and analyzed for the indexes specified in Section 2.2.2. The results showed that the accuracy of the six experiments was within 1.78%, the SNR was greater than 1245.87, and the analysis time was within 19.76 min, which demonstrated a robust MODR.

##### Linearity, Accuracy Investigation, and Calculation of LOD and LOQ

Seven samples with total DTSs concentrations of 9, 5, 2.5, 1.5, 1.0, 0.5, and 0.25 mmol/L were obtained by gradient dilution. The seven samples were analyzed according to the method described in Section 2.3.1. A linear regression was performed with the actual concentration prepared as “y” and the measured concentration as “x”. The regression equation was obtained as *y* = 0.9968*x* + 0.0472, with a coefficient (r^2^) of 0.9999, demonstrating the excellent linearity of the method. The slope of the equation was close to 1.00, indicating good accuracy. Based on the equation, the LOD and LOQ were calculated as described in Section 3.6 and the results were LOD = 0.0794 mmol/L and LOQ = 0.2647 mmol/L. The recovery rates of the six samples with total DTSs content above LOQ were calculated. The results showed that the recoveries of all six samples were in the range of 98.87~102.89%, which met the ATP requirements.

##### Precision and Sample Stability Investigation

Intra-day and inter-day precision were investigated by repeated experiments, and relative standard deviations (RSDs) of six parallel experiments were 0.67% and 1.14%, respectively. A sample was placed at room temperature and analyzed three times at 0 h and 48 h, respectively, to examine the stability within 48 h. The results show that the RSD of the 6 experiments was 1.81%. All these results met the ATP requirements and proved that the intra-day precision, inter-day precision, and 48 h stability of the samples were good.

##### Signal Specificity Investigation

According to the structural information provided by the NMR spectrum, the quantitative signals and the internal standard signals were highly specific in the absence of overlapping and interfering impurity signals. Since the samples in method development were prepared with standards, impurity interference could be excluded. Signal overlapping may exist when the method is applied to actual TCM samples. Therefore, quantitative signal specificity needs to be examined at the time of method transfer for each type of sample.

##### Measurement Uncertainty

**Figure 4:**
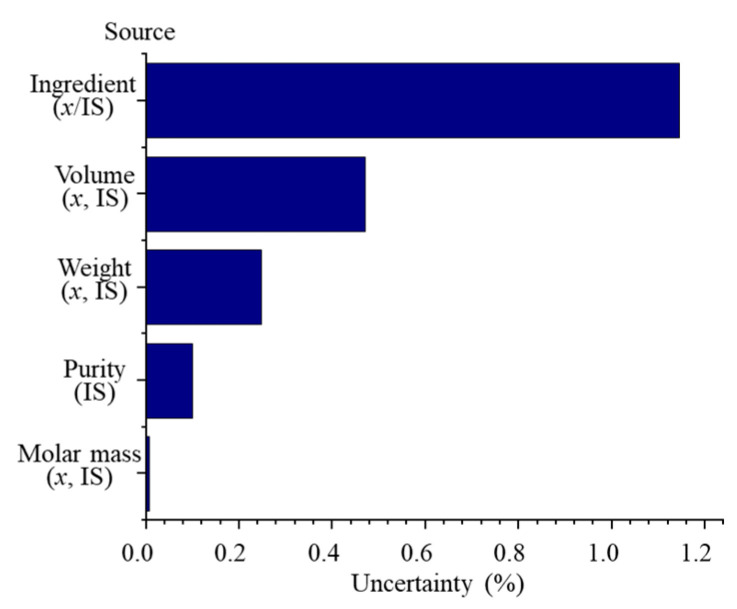
Contribution of different sources of errors to the combined uncertainty.

The combined uncertainty (CU) and the expanded uncertainty (EU) for the total DTSs ^1^H qNMR method were calculated from Equations (S2) and (S3). The results were 1.21% and 2.42%, respectively, which met the ATP requirements. CU was the combination of uncertainties from the molecular weight of the internal standard and DTSs, the purity of the internal standard, the weighing measurement process, the volume measurement process, and the integral operation. The contributions of the uncertainties from different sources are shown in Figure 4. The most significant contribution was the integration result error, followed by the volume measurement error.

### 2.4. CPPV Example: Method Transfer and Application

Traditional method transfer strategies include comparative testing, method co-validation, method re-validation, and transfer exemptions. In the APLC concept, the transfer of analytical methods can be considered part of the method performance validation. In this study, the transfer and application of the ^1^H qNMR method were performed on three samples of herbal medicines containing DTSs. The three samples included a liquid preparation (Shenmai Injection), a solid preparation (Xuesaitong Injection), and a process intermediate (Gynostemma column chromatography eluting intermediates). For the ^1^H qNMR method in this study, the conditions of reagents and instruments were unchanged. When the method was transferred to the determination of different kinds of samples, the most significant issue was signal overlapping of impurity signals, which affected the method’s accuracy. Therefore, the transfer of the method required revalidation of the specificity of the quantitative signals and the accuracy of the results, while other methodological indexes did not need to be revalidated. Given that the signals corresponding to both H-26 and H-27 protons can independently serve as quantitative peaks, the method remains valid even if one signal experiences interference while the other remains unaffected. The specificity of the internal standard signals of the above three samples has been verified by 2D NMR experiments. ^1^H-^13^C HSQC and ^1^H-^1^H COSY results both showed that the internal standard signals were independent. The quantitative signals were the overlapping signals of multiple saponins so their specificity could not be verified simply by 2D experiments, which were confirmed by negative control experiments in this study.

#### 2.4.1. ^1^H qNMR Analysis of Total Dammarane-Type Ginsenosides in the Shenmai Injection 

Shenmai Injection is an herbal preparation with ginsenoside as API, prepared from *red ginseng* and *Ophiopogon japonicus.* In total, 600 µL of Shenmai Injection was measured and lyophilized to volatilize the solvent completely. Then, 600 µL of CD_3_OD solution containing DMT was added to dissolve the remaining solid, and the supernatant was centrifuged at 10,000 rpm for 10 min to obtain the Shenmai Injection NMR sample.

The D101 type macroporous resin was activated by soaking in ethanol for 24 h, mounted on a column (1.5 cm × 12 cm), and washed with deionized water until there was no alcohol smell. In total, 1 mL Shenmai Injection was added to the column and eluted with 25 mL of deionized water at a flow rate of 0.5 mL/min. The eluate was collected, at which time the DTSs were retained in the macroporous resin column. After completely evaporating the solvent of the eluent, 600 µL of CD_3_OD containing DMT was added to re-dissolve the remaining solid. Then, the mixture was centrifuged at 10,000 rpm for 10 min to get supernatant as the negative control sample A of Shenmai Injection.

In addition, to exclude the influence of *Ophiopogon japonicus*-related components on the quantitative signals, the *Ophiopogon japonicus*-related process intermediate “*Ophiopogon japonicus* aqueous supernatant” from the production of Shenmai Injection was taken and the sample was prepared in the same way as that of Shenmai Injection to obtain the negative control sample B.

**Figure 5:**
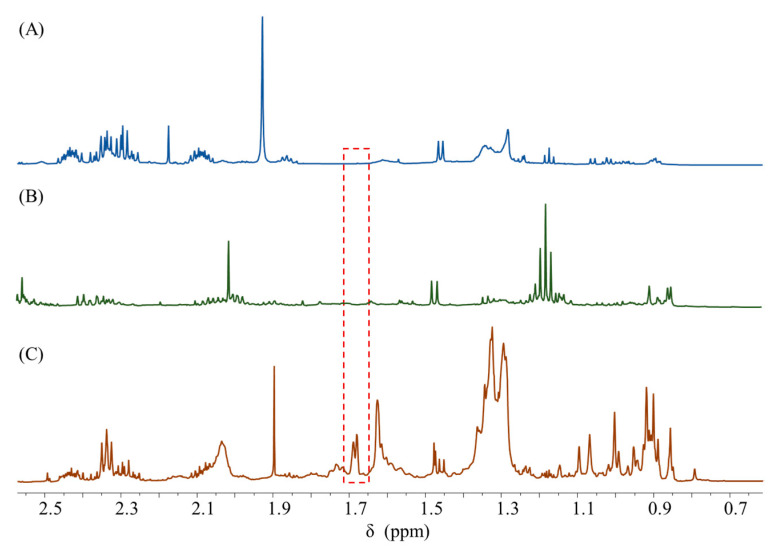
^1^H NMR spectra of (**A**) negative control sample A, (**B**) negative control sample B, and (**C**) Shenmai Injection sample. The red box indicates the location of a quantitative peaks.

The ^1^H NMR spectra of Shenmai Injection, negative control samples A and B were collected by the method described in Section 2.3.1, and the results are shown in Figure 5. The two negative control samples showed no signal response at 1.68 ppm, which proved that the quantitative peak specificity at 1.68 ppm was good.

After that, the accuracy of the method was investigated by recovery experiments. Three groups of samples were prepared with standard addition amounts of 80%, 100%, and 120%, respectively. The spectra of the above samples were collected to calculate the recovery rates. The average recovery rates were 98.95%, 99.58%, and 100.84% for the low, medium, and high concentration groups, respectively, indicating that ^1^H qNMR could accurately quantify the total DTSs in the Shenmai Injection.

#### 2.4.2. ^1^H qNMR Analysis of Total Notoginsenosides in the Xuesaitong Injection 

In total, 5.29 mg of Xuesaitong Injection was weighed precisely in a centrifuge tube, and 600 µL of CD_3_OD containing DMT was added and dissolved to obtain the Xuesaitong NMR sample. Xuesaitong Injection is purified notoginsenoside, the DTSs of which accounted for more than 90% of the total solids. Therefore, the ^1^H qNMR spectrum Xuesaitong Injection was similar to the samples described in Section 2.2.1, and the interference of impurity signals could be excluded. The signals showed high specificity in the spectrum.

The accuracy of the method was also investigated by recovery experiments. The recovery experiments procedure was similar to that in Section 2.4.1. The average recovery rates of the three concentration groups were 100.62%, 98.14%, and 99.63%, respectively, which demonstrated that ^1^H qNMR could accurately quantify the total notoginsenosides in the Xuesaitong Injection.

#### 2.4.3. ^1^H qNMR Analysis of Total Gypenosides in the Gynostemma Process Intermediates 

**Figure 6:**
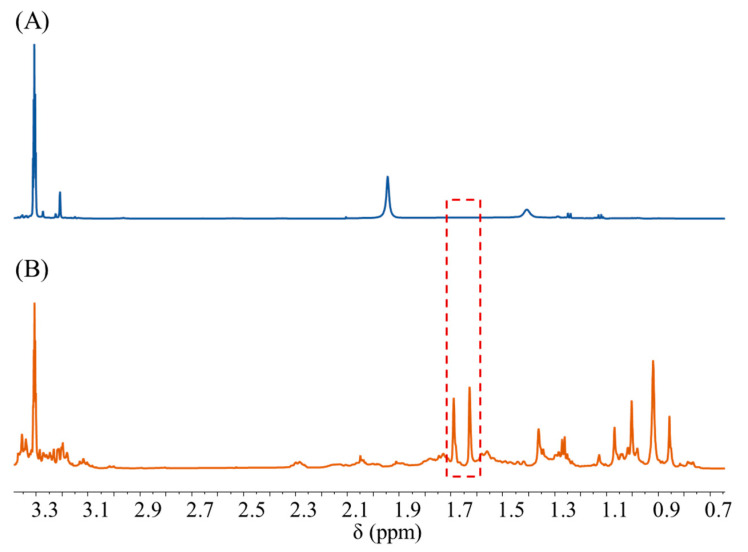
^1^H NMR spectra of (**A**) the negative control sample and (**B**) the positive control sample of gypenosides. The red box indicates the location of a quantitative peaks.

The 70% ethanol eluting intermediate of gynostemma column chromatography was precisely pipetted into a centrifuge tube and concentrated by centrifugation until the water evaporated, after which 600 µL of CD_3_OD containing DMT was added to dissolve the remaining solid, and the mixture was centrifuged at 10,000× *g* rpm for 10 min to obtain the supernatant as gynostemma NMR sample. The negative sample was prepared in the same way on water eluting intermediate of gynostemma column chromatography. The NMR spectra of the gynostemma NMR sample and the negative sample were obtained by the method described in Section 2.3.1. The results are shown in Figure 6, and the negative sample showed no signal response at 1.68 ppm and 1.62 ppm, which proved the excellent specificity of the quantitative signals at both locations.

The accuracy was verified by comparison with an independent method of known authenticity. Six batches of gynostemma column chromatography eluting intermediates were analyzed by applying the classical glacial acetic acid-perchloric acid UV chromogenic method and ^1^H qNMR method respectively to determine total gypenosides. Table 5 presents the outcomes obtained from the utilization of both methods, indicating that the relative deviations of two methods fall within the permissible range of 5%. Paired t-tests were performed on the results for the two groups of measurements. The 95% confidence interval for the deviation of the results between the two methods was (−1.690, 0.409) with a test *p*-value of 0.178 (>0.05), which proved that there was no significant difference between the two groups of measurements. Both the results of relative deviations and paired t-test demonstrated that the ^1^H qNMR method was in high agreement with the analytical results of independent methods of known authenticity, i.e., ^1^H qNMR can accurately quantify the content of total gypenosides in gynostemma process intermediates. Notwithstanding, the ^1^H NMR technique exhibits a superior level of operational ease, environmental sustainability, and efficiency in contrast to traditional methodologies.

## 3. Materials and Methods

### 3.1. Reagents and Materials

Deuterated methanol containing 0.03% *v*/*v* tetramethylsilane (TMS) (CD_3_OD, 99.8% D) was purchased from Cambridge Isotope Laboratories, Inc. (Tewksbury, Massachusetts, USA); dimethyl terephthalate (DMT, Lot No. BCBF6171V) was purchased from Sigma-Aldrich (Darmstadt, Germany); standard substances Ginsenoside Rb1 (Lot No. 200725), Ginsenoside Rg1 (Lot No. 200709), Ginsenoside Re (Lot No. 200603), Panaxoside R1 (Lot No. 190813), Panaxoside Fe (Lot No. 190803), Gynostemma saponin XLIX (Lot No. 210826), Gynostemma saponin XVII (Lot No. 210903), and Gynostemma saponin XLVI (Lot No. 210730) were purchased from Shanghai Winherb Medical Technology Co., Ltd. (Shanghai, China); Shenmai Injection was provided by Chiatai Qingchunbao Pharmaceutical Co., Ltd. (China); Xuesaitong Injection was provided by Heilongjiang ZBD Pharmaceutical Group Co., Ltd. (Hangzhou, China); and gynostemma column chromatography eluting intermediate was provided by Wanbangde Pharmaceutical Group Co., Ltd. (Taizhou, China).

### 3.2. Primary Analysis Conditions

In this study, the internal standard method was used to quantify total DTSs. Before screening and optimization of CAPPs, some primary analytical conditions need to be initially confirmed, including deuterated reagents, pulse sequences, internal standard substances, and signals for quantification.

#### 3.2.1. Selection of Deuterated Reagents

In NMR-related studies of DTSs, commonly used deuterated reagents include deuterated pyridine (C_5_D_5_N) and deuterated methanol (CD_3_OD). Both solvents can better solubilize DTSs, but the signal resolution in CD_3_OD is much higher than that in C_5_D_5_N. Therefore, CD_3_OD was chosen as the deuterated reagent in this study.

#### 3.2.2. Selection of Pulse Sequences

The ^1^H NMR spectrum acquisition with CD_3_OD as the solvent can be completed directly with a standard hydrogen pulse ZG30. However, the water signal in the spectrum acquired by ZG30 is high, which may affect the sensitivity of signals for quantification, as shown in Figure S1. Therefore, the pre-saturated water suppression pulse NOESYGPPR1D was considered. This study compared the average signal-to-noise ratio (SNR) of the same signals in the spectra acquired under the NOESYGPPR1D and ZG30 pulses. The SNR of the signal acquired under the NOESYGPPR1D pulse was significantly higher than that of the signal acquired under the ZG30 pulse, and the higher percentage was more than 50%, indicating that the use of NOESYGPPR1D pulse has a more remarkable improvement in sensitivity, so this pulse was chosen in this study. The specific experimental results are shown in Section S1.

#### 3.2.3. Selection of Internal Standard

The requirements for the internal standard are high solubility, and good stability without signal overlapping in the sample. DMT was chosen as the internal standard in this study because the spin-lattice relaxation time (T_1_) of the protons of DMT in CD_3_OD is relatively short, which can support a shorter analysis time. In the ^1^H NMR spectrum, DMT has two signals at 3.94 ppm and 8.11 ppm, respectively. The singlet of -O-CH_3_ at 3.94 ppm tends to overlap with the other signals in the sample, so the singlet of -CH at 8.11 ppm was used for quantification, and the number of equivalent protons for this signal was four.

#### 3.2.4. Selection of Signals for Quantification

**Figure 7:**
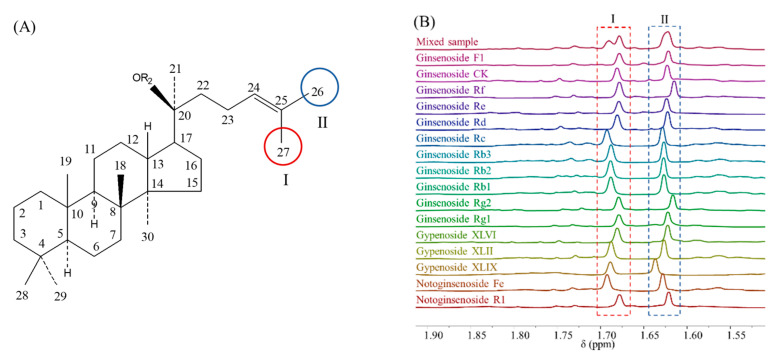
(**A**) Structure of dammarane-type tetracyclic triterpenes; (**B**) Signals for quantification of total DTSs.

The structural skeleton of DTSs is shown in Figure 7A. The H-26 and H-27 in the side chain of the methyl structure are in almost all DTSs. Although some side chain modifications of DTSs, such as cyclization, double bond transfer, etc., have been reported in recent years, the saponins with the above conditions belong to trace components, accounting for less than 1% of the total content of DTSs [2]. The effect of these saponins is negligible for the macroscopic index of “total DTSs” content. The H-26 and H-27 protons signals in the ^1^H NMR spectrum are at 1.62 ppm and 1.68 ppm, which are the signal I and II labeled in Figure 7B.

In a mixture of multiple DTSs, H-26 and H-27 signals of different DTSs overlap and stack at the positions of signal I and II, respectively, so the areas of these two signals contain the information of total DTSs content and the number of equivalent protons for both sets of peaks is three. In summary, signal I and II can be used individually or in combination as the signals for quantification of total DTSs; and their areas can be used to calculate the exact molar concentration of total DTSs in the sample, as shown in Equation (4):

(4) $$M_{TS}=\frac{N_{DMT}\cdot A_{TS}}{N_{TS}\cdot A_{DMT}}\cdot M_{DMT}$$

where $M_{TS}$ and $M_{DMT}$ are the molar concentrations of total DTSs and the internal standard (DMT), respectively, $A_{TS}$ and $A_{DMT}$ are the integrated areas of signals of total DTSs and DMT, respectively, and $N_{TS}$ and $N_{DMT}$ are the number of equivalent protons for signals of DTSs and DMT, respectively.

#### 3.2.5. Data Processing

After spectra acquisition, Fourier transform and manual phase correction of the free induction decay (FID) signals were performed in Topspin software (version 3.2.6), and the chemical shift correction was performed with the TMS peak (0.00 ppm) as reference. The window function was selected in exponential mode and the line width (LB) was set as 0.3 Hz. Afterward, the signals of DMT and total DTSs were integrated by “line fitting” in MestReNova software (version 14.0.0), and the molar concentrations of total DTSs were calculated according to Equation (1).

### 3.3. Determination of ATPs

The prospective requirements of the qNMR method in this study were well defined in the ATPs, which included all elements such as target sample, target APIs, sample preparation, required analytical technique, method requirement, target application, reportable quality attributes, and critical analytical characteristics. Further information regarding the aforementioned details can be found in Section S2.

### 3.4. Risk Assessment Methodology

The main tools used to perform risk assessment in this study were the Ishikawa diagram and FMECA. Ishikawa diagram was used for risk identification, but it cannot determine the correlation between parameters. Therefore, FMECA was introduced as a risk-ranking method in this study. The risk priority number (RPN) is determined by the severity (S), probability of occurrence (O), and ease of detection (D) of the failure factor. The higher the RPN score, the higher the influence of the factor on the analysis results, which needs to be focused on in the process of method development [12].

### 3.5. DoE Methods

The purpose of DoE is to establish an experimental program to obtain “maximum information” through statistical methods with “minimum experimentation”. The statistical models are used in DoE to help researchers understand the impact of APPs on method performance and provide reliable estimates of analytical method detection. This study used PBD to further screen the high-risk parameters. The screened CAPPs were optimized by CCD, which is suitable for fitting multivariate non-linear models, ensuring the model’s fit without increasing the number of runs. Based on the optimized model established by CCD, the design space was calculated according to the ATPs requirements and MODR was selected. Design of the experiments, statistical modeling, and design space calculation in this study were performed on MODDE 13 software (Umetrics Inc., Sartorius AG, Göttingen, Germany).

### 3.6. Indexes of APPQ

Based on the requirements in the ATPs, accuracy, precision, specificity, limit of detection (LOD), limit of quantification (LOQ), linearity, robustness, and measurement uncertainty were determined with reference to ICH Q2 (R1) guideline, the specific validation process is shown in Section S3.

## 4. Conclusions

In this study, a ^1^H qNMR method for the determination of total DTSs in herbal medicines was developed based on APLC. In the initial stage of APLC (APD), the Ishikawa diagram and FMECA were applied to identify the potential risk factors and select seven parameters as potential CAPPs. DoE methods were used to screen and optimize the CAPPs, and a robust MODR was obtained through modeling. In the subsequent stage (APPQ), all the methodological indexes performed well; the combined uncertainty and the expanded uncertainty for the ^1^H qNMR method were 1.21% and 2.42%, respectively. Finally, the developed ^1^H qNMR method was transferred and successfully applied to the quantitative analysis of total DTSs in three herbal medicines, providing an example of CPPV. It should be noted that the APLC has yet to be completed, and routine monitoring of the method should be carried out during subsequent applications. Timely investigation as well as corrective and preventive measures should be taken if there is any indication that the analytical method is out of control.

In comparison to the conventional UV colorimetric method, the ^1^H qNMR method developed in this study offers several advantages, including a smaller required sampling amount, simplified sample pretreatment procedures, and avoidance of corrosive reagents. Significantly, the total DTSs content determined via this methodology represents the true value as measured by molar quantity, in contrast to the estimated value derived from the traditional approach. Therefore, the ^1^H qNMR method provides a higher degree of accuracy. The method development process applied QRM and AQbD concepts, allowing for a comprehensive examination of various factors. It effectively minimized the potential for errors and omissions while optimizing parameter efficiency. In addition, the utilization of models facilitated a quantitative assessment of multiparameter effects and their interactions.

## Figures and Tables

**Table 1:** Experimental design table for PBD screening and coding values of each factor.

NO.	*x*_1_ (mmol/L)	*x*_2_ (Times)	*x*_3_ (Times)	*x*_4_ (pcs)	*x*_5_	*x*_6_ (Seconds)	*x*_7_ (K)
1	5 (+1)	0 (−1)	128 (+1)	16,384 (−1)	40.3 (−1)	2 (−1)	300 (+1)
2	5	8 (+1)	16 (−1)	65,536 (+1)	40.3	2	296 (−1)
3	0.5 (−1)	8	128	16,384	161 (+1)	2	296
4	5	0	128	65,536	40.3	30 (+1)	296
5	5	8	16	65,536	161	2	300
6	5	8	128	16,384	161	30	296
7	0.5	8	128	65,536	40.3	30	300
8	0.5	0	128	65,536	161	2	300
9	0.5	0	16	65,536	161	30	296
10	5	0	16	16,384	161	30	300
11	0.5	8	16	16,384	40.3	30	300
12	0.5	0	16	16,384	40.3	2	296
13	2 (0)	4 (0)	64 (0)	32,768 (0)	90.5 (0)	10 (0)	298 (0)
14	2	4	64	32,768	90.5	10	298
15	2	4	64	32,768	90.5	10	298

**Table 2:** Results of evaluation indexes in PBD experiments.

NO.	Accuracy (%)	SNR	Resolution
1	77.07	2446.8	0.71
2	17.65	1316.0	0.62
3	55.75	1601.0	1.35
4	0.291	4174.4	0.68
5	27.69	1343.4	0.61
6	0.709	2771.8	1.29
7	0.411	3407.8	0.96
8	23.07	3404.6	0.75
9	0.747	1660.5	0.72
10	9.572	1589.5	0.72
11	4.133	965.6	1.36
12	51.68	658.6	1.15
13	6.202	3288.7	0.63
14	6.055	3451.9	0.60
15	6.263	3318.1	0.60

**Table 3:** Parameters setting and evaluation index results of the CCD experiments.

NO.	Parameter Setting			Evaluation Indexes		
	X_1_ (Times)	X_2_ (pcs)	X_3_ (Seconds)	Accuracy (%)	SNR	T_q_ (Minutes)
1	32 (−1)	32,768 (−1)	20 (−1)	1.742	1314.49	13.68
2	32	98,304 (+1)	20.00	1.327	1270.59	13.68
3	80 (+1)	32,768	20.00	1.567	1904.43	31.93
4	80	98,304	20.00	0.947	1908.12	31.93
5	32	32,768	30.00 (+1)	0.785	1225.88	19.68
6	32	98,304	30.00	0.396	1217.23	19.68
7	80	32,768	30.00	0.186	2006.74	45.93
8	80	98,304	30.00	0.037	2010.88	45.93
9	56	16,384 (−α)	25.00	2.161	1621.74	27.80
10	56	131,072 (+α)	25.00	0.066	1678.88	27.80
11	16 (−α)	65,536	25.00	1.238	843.20	9.27
12	96 (+α)	65,536	25.00	0.130	2215.89	46.35
13	56	65,536	16.59 (−α)	1.830	1689.57	19.40
14	56	65,536	33.41 (+α)	0.049	1609.00	36.22
15	56 (0)	65,536 (0)	25.00 (0)	0.723	1577.34	27.80
16	56	65,536	25.00	0.623	1637.77	27.80
17	56	65,536	25.00	0.718	1652.33	27.80

**Table 4:** Regression coefficient, r^2^, and r^2^_adj_ of PLS models of evaluation index for CCD experiments.

Parameter Items	Regression Coefficient		
	Accuracy (%)	SNR	T_q_ (Minutes)
*b*_0_	0.855	1633.050	27.802
X_1_	−0.228	345.696	10.240
X_2_	−0.338	-	-
X_3_	−0.485	−4.90011	4.619
X_1_^2^	-	−29.765	0.002
X_3_^2^	-	-	0.002
X X_13_	-	36.901	1.701
r^2^	0.853	0.988	1.000
r^2^_adj_	0.819	0.983	1.000

Note: Parameters that are not significant in all three models are not listed.

**Table 5:** Comparison of the ultraviolet colorimetric method and ^1^H qNMR method.

No.	UV (mmol/L)	^1^H qNMR (mmol/L)	Relative Deviation (%)
1	36.45	36.53	−0.21
2	39.56	41.12	−3.78
3	43.07	42.26	1.92
4	40.86	41.80	−2.25
5	38.94	40.80	−4.55
6	36.45	36.68	−0.61
Mean values	39.22	39.86	−1.61

## Data Availability

The data underlying this article are available in the article and in its online supplementary material.

## Supplementary Materials

The following supporting information can be downloaded at: https://www.mdpi.com/article/10.3390/ph16070947/s1, Section S1: Determination of the acquisition pulse; Section S2: Determination of ATPs; Section S3: Process performance validation; Section S4: Risk identification and assessment results based on Ishikawa diagram and FMECA; Figure S1: Results of SNR paired *t*-test for signals collected under two pulses.

## Abbreviations

DTSs: dammarane-type saponins; ^1^H NMR: proton nuclear magnetic resonance; ^1^H qNMR: proton quantitative nuclear magnetic resonance; APIs: active pharmaceutical ingredients; APLC: analytical procedure lifecycle; ATP: analytical target profile; APD: analytical procedure design; APPQ: procedure performance qualification; CPPV: continued procedure performance verification; QRM: quality risk management; DoE: design of experiments; CAPPs: critical analytical procedure parameters; MODR: method operable design region; AQbD: analytical quality-by-design; FMECA: failure mode effects and criticality analysis; PBD: Plackett–Burman design; CCD: central composite design; TMS: tetramethylsilane; DMT: dimethyl terephthalate; SNR: signal-to-noise ratio; RPN: risk priority number; LOD: limit of detection; LOQ: limit of quantification; DS: number of dummy scans; NS: number of scans; TD size of fid; RG: receiver gain; D_1_: relaxation delay; SW: pulse width; P_1_: 90° pulse width; T_l_: time lag; CU: combined uncertainty; EU: expanded uncertainty.
