# Supplementary material for: Application of the Analytical Procedure Lifecycle Concept to a Quantitative 1H NMR Method for Total Dammarane-Type Saponins
Source: Pharmaceuticals (Basel). 2023 Jun 29;16(7):947. doi: 10.3390/ph16070947 (PMC10383815; doi:10.3390/ph16070947)
Supplement: Supplementary file 1 [file pharmaceuticals-16-00947-s001.zip › pharmaceuticals-2448903-supplementary.pdf]

Supplementary Material

Title: Application of the analytical procedure lifecycle concept to a quantitative ^{1}H NMR method for total dammarane-type saponins

Authors: Wenzhu Li[1,2#], Jiayu Yang[1,2#], Fang Zhao[1,2], Xinyuan Xie[1,2], Jianyang Pan[1,2], Haibin Qu[*1,2]

Contents

Section S1. Determination of the acquisition pulse S2

Table S1 Results of SNR paired t-test for signals collected under two pulses S2

Figure S1 Comparison of ^{1}H NMR spectra acquired with pulsed NOESYGPPR1D (A), and ZG30 (B) S3

Section S2. Determination of ATPs S3

S2.1. Target samples and API S3

S2.2. Sample preparation requirements S3

S2.3. Target application and methodological requirements S3

S2.4. Reportable quality attributes and critical analytical characteristics S4

Section S3. Process performance validation S4

Section S4. Risk identification and assessment results based on Ishikawa diagram and FMECA S6

Table S2 Definitions of failure severity (S), failure occurrence (O) and failure detection (D) for ^{1}H qNMR method........ S7

Table S3 Risk ranking of ^{1}H qNMR method based on FMECA S8

Section S1. Determination of the acquisition pulse

To verify the influence of the water signal on the SNR of the quantitative signal, 10 Dammarane-type saponins (DTSs) samples were acquired with pulses ZG30 and NOESYGPPR1D. Other analytical parameters were kept consistent to calculate the difference in SNR between the corresponding signals under both pulses. Five groups of representative signals were selected from the spectra acquired under the two pulses. The SNRs and the mean relative deviations (MRDs) between the corresponding signals were calculated separately for each group of signals in spectra. Then, paired t-tests were performed, and the results were shown in Table S1. The formula for MRDs is shown in Equation S1, where MRD_i is the MRD of the ith signal, the $\overline{SNR}_{iN}$ and $\overline{SNR}_{iZ}$ were the average SNR of the ith signal under NOESYGPPR1D and ZG30 pulses, respectively.

$$MRD_i = \frac{(\overline{SNR}_{iN} - \overline{SNR}_{iZ})}{\overline{SNR}_{iZ}} \times 100\% \qquad \text{Equation S1}$$

Table S1. Results of SNR paired t-test for signals collected under two pulses

Signal	Pulse	SNR mean value	Standard error	T-value	P-value	MRD (%)
Signal 1	ZG30	176.7	16.0	-5.49	0.00	51.61
	NOESYGPPR1D	267.9	24.8			
Signal 2	ZG30	520.2	19.3	-17.16	0.00	74.22
	NOESYGPPR1D	906.3	23.3			
Signal 3	ZG30	126.2	10.0	-9.90	0.00	62.12
	NOESYGPPR1D	204.6	17.4			
Signal 4	ZG30	390.0	16.4	-16.17	0.00	68.54
	NOESYGPPR1D	657.3	15.3			
Signal 5	ZG30	261.9	13.7	-18.48	0.00	76.02
	NOESYGPPR1D	461.0	10.4			

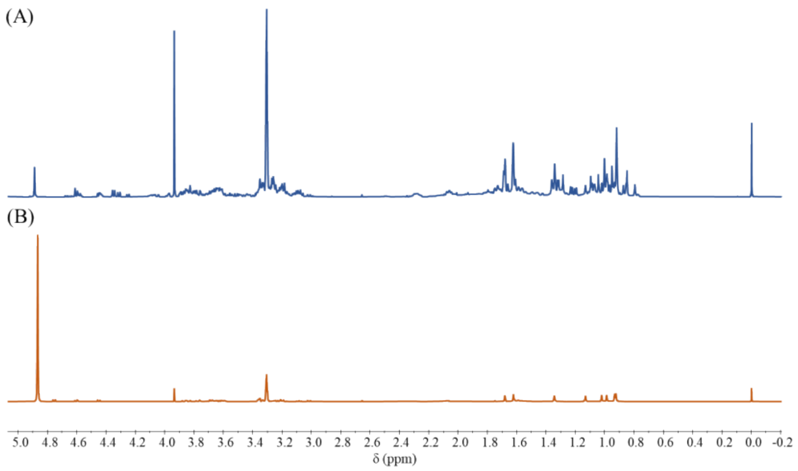

Figure S1 Comparison of ^{1}H NMR spectra acquired with pulsed NOESYGPPR1D (A), and ZG30 (B)

Section S2. Determination of ATPs

S2.1.Target samples and API

The target API in this study was total DTSs, so the target analysis was performed on herbal medicines-related preparations and process intermediates with DTSs as the main active ingredient, such as extracts of *ginseng*, *Panax ginseng*, *gynostemma,* and other preparations with these herbs as the main raw materials.

S2.2. Sample preparation requirements

The preparation requirements for solid or liquid sample were different. For solid samples, the samples were prepared by direct weighing and dissolving in the deuterated solvent containing internal standard. For liquid samples, the original solvent was evaporated by freeze-drying (LABCONCO freeze dryer, USA) or centrifugal concentration (Vacuum centrifugal evaporation concentrator, Model SPD121P-230 with RVT4104 refrigeration steam trap, Thermo Electron, USA) and re-dissolved by adding deuterated solvent containing internal standard. Then the re-dissolved solution was centrifuged (5425 high-speed centrifuge, Eppendorf, Germany) at 10,000 rpm for 10 minutes to obtain the supernatant for analysis.

S2.3. Target application and methodological requirements

The target application of this study was absolute quantitative analysis, so the most essential requirement for the method was accurate and reliable quantitative results. In this study, Analytical Procedure Parameters (APPs) that affect the performance quality of ^{1}H qNMR analysis were required to investigate,

screen and optimize during the method development. Besides, in order to reflect the advantages of analysis speed, the duration of analysis was expected to be as short as possible on the premise of ensuring the method's robustness, with a maximum of 25 minutes.

S2.4. Reportable quality attributes and critical analytical characteristics

Reportable quality attributes and critical analytical characteristics can be defined in various ways, while accuracy and precision are among the most important components. In this study, an upper limit of 3% was given for accuracy bias and precision of reported values, i.e., accuracy was required to be within 100% ± 3% and precision less than 3%. In addition, measurement uncertainty was usually used as a synthetic index of accuracy and precision, and this study requires the measurement uncertainty of the reported values to be less than 5%.

Section S3. Process performance validation

Based on the requirements in the ATPs, the following indexes were determined with reference to ICH Q2 (R1) guideline.

Accuracy: Accuracy is considered in order to avoid systematic errors in the measurement method. In this study, proof of accuracy can be accomplished through recovery experiments, or comparison with an independent method with known accuracy.

Precision: The precision of qNMR is investigated in a similar way to that of chromatography, in which the same or similar objects are measured repeatedly under specified conditions and the agreement of the multiple measurements is compared. Relative Standard Deviation (RSD) is usually used as the precision index. In this study, intra-day precision (repeated measurements within a short period of time on the same date) and inter-day precision (repeated measurements in different dates) were investigated.

Specificity: NMR spectra provide very detailed structural information and quantitative signals, which are in principle highly specific. However, in complex systems such as herbal medicines, signal overlapping may lead to unachievable specificity. Therefore, in this study, the specificity of the quantitative signals can be demonstrated by preparing a negative sample of DTSs by column chromatograph.

Limit of Detection (LOD) and Limit of Quantification (LOQ): LOD is related to the identification, and LOD is related to the quantification. The equations are, LOD = $3.3\sigma/S$ and LOD = $10\sigma/S$, where σ refers

to the deviation from the Y-axis intercept (non-zero) of the linear regression curve and S refers to the slope of the linear regression curve.

Linearity range: Due to the high dynamic range of modern NMR spectrometers, ^{1}H qNMR can measure a wide range of concentrations, usually starting from the LOQ (lower limit) to the solubility of the analyte (upper limit). In principle, the correlation between concentration and peak area is consistent with linearity, but overloads should also be considered. Therefore, seven concentration gradients were used in this study to assess linearity.

Robustness: Based on the AQbD concept, the robustness of the analytical results can be ensured by operating in MODR, however, non-parametric factors such as degradation of the analyte at room temperature have to be considered. In this study, the stability of DTSs was investigated over 48 hours.

Measurement uncertainty: In this study, the measurement uncertainty of ^{1}H qNMR method is expressed as a combination of uncertainty from molar mass, weighing, IS purity, and integral values, also known as Combined Uncertainty (CU), which is calculated according to Equation S2.

$$CU = \sqrt{\frac{u(M_x)}{M_x} + \frac{u(M_{IS})}{M_{IS}} + \frac{u(P_{IS})}{P_{IS}} + \frac{u(W_{IS})}{W_{IS}} + \frac{u(W_x)}{W_x} + \frac{u(V_x)}{V_x} + \frac{u(I_x/I_{IS})}{I_x/I_{IS}}} \quad \text{Equation S2}$$

where $u(M_x)\ /M_x$ and $u(M_{IS})\ /M_{IS}$ are the uncertainty components from the molar mass of ingredient x and IS, respectively, which can be calculated according to the values given by IUPAC; $u(P_{IS})\ /P_{IS}$ is the uncertainty component from the purity of IS, which is given by IS standard certificate; $u(W_x)\ /W_x$ and $u(W_{IS})\ /W_{IS}$ are the uncertainties from the weighing process of ingredient x and the IS, respectively, given by the uncertainty parameters of the used balance (provided by the manufacturer); $u(V_x)\ /V_x$ is the uncertainty from the volume measurement during the sample preparation, given by the uncertainty parameters of the used pipettors (provided by the manufacturer). $u(I_x/I_{IS})\ /I_x/I_{IS}$ is the uncertainty component from the integral result of a characteristic peak, which is given by the RSDs of precision investigation. To satisfy the 95% confidence interval, the Extended Uncertainty (EU) was calculated using a factor k = 2, as shown in Equation 3.

$$EU = CU \times 2 \quad \text{Equation S3}$$

Section S4. Risk identification and assessment results based on Ishikawa diagram and FMECA

In this study, the risk factors that may affect the results of ^{1}H qNMR analysis as potential failure factors of FMECA were dissected from five categories: Sample preparation, acquisition, data processing, environment and instrument. The factors from sample preparation include sample preparation method (sample concentration, loading volume, NMR tube quality), operation error (operator error, gauge error), and sample storage (storage temperature, storage time). The factors from spectra acquisition are mainly the acquisition parameters, including number of scans (NS), number of dummy scans (DS), size of fid (TD), spectral width (SW), sampling temperature, mixing time (D_8), transmitter frequency offset (O_1), relaxation delay (D_1), receiver gain (RG), etc. The factors from data processing are data processing parameters, including time-domain data processing parameters (zero filling, window function) and frequency-domain data processing parameters (phase correction, baseline correction, integration method). The factors from environment can be divided into sample preparation environment and spectra acquisition environment, mainly including temperature, humidity, air pressure and other environmental factors. The factors from the instrumentation include magnetic field uniformity and equipment stability, etc.

FMECA is used to evaluate risk factors identified by the Ishikawa diagram in terms of failure severity (S), probability of occurrence (O) and ease of detection (D). Severity (S) assesses the impact of a potential failure mode on the analysis results when it occurs, and is evaluated on a scale of 1-5 in this study, with 1 being almost no impact and 5 being a very serious impact. Occurrence probability (O) refers to the chance of a specific failure occurring and is evaluated on a scale of 1-5 in this study, with 1 being an unlikely or low occurrence of failure and 5 being a high occurrence of failure. Detection level (D) is the evaluation index of the possibility to find out the failure or the cause of the failure, the evaluation level in this study is 1-5, where 1 means the failure can be directly detected or visually found, and 5 means the failure is difficult to be directly detected and requires in-depth inspection of the equipment. The definitions of failure severity (S), failure occurrence (O) and failure detection (D) for ^{1}H qNMR method are shown in Table S2.

Table S2 Definitions of failure severity (S), failure occurrence (O) and failure detection (D) for ^{1}H qNMR method

Severity (S)	Severity Definition	Rating Level	Probability of occurrence (O)	Probability of occurrence definition	Rating Level	Detection level (D)	Test level definition	Rating Level
Class I	Causes complete distortion of the spectrum, which seriously affects the accuracy and precision of the method	5	Class I	Very high probability of occurrence and difficult to avoid	5	Class I	Data processing and calculations are required to detect and determine failure modes	5
Class II	Causes a significant decrease in spectral resolution or signal-to-noise ratio, and has a more serious impact on the accuracy or precision of some peak integration results	4	Class II	Happens often	4			
Class III	Causes distortion of some spectral signals, which affects the accuracy or precision of some peak integration results to some extent	3	Class III	There is a certain possibility of occurrence	3	Class II	Failure modes can be determined by visual inspection or simple comparison at the end of mapping acquisition	3
Class IV	Causes some degree of spectral resolution or signal-to-noise ratio reduction, which may have a small effect on the accuracy or precision of some peak integration results	2	Class IV	Low probability of occurrence, or not yet occurring but theoretically possible	2			
Class V	No or minimal impact	1	Class V	Theoretically less prone to failure, or failure can be avoided	1	Class III	Failure modes can be determined at the sample preparation stage or during spectrum acquisition	1

Table S3 Risk ranking of ^{1}H qNMR method based on FMECA

Category	Serial number	Factors	S	O	D	RPN
Acquisition	1	NS	5	5	5	125
Acquisition	2	RG	5	5	5	125
Acquisition	3	D_1	5	5	5	125
Acquisition	4	TD	4	5	5	100
Sample Preparation	5	Sample concentration	3	5	5	75
Acquisition	6	DS	3	5	5	75
Acquisition	7	Temperature	3	5	5	75
Data processing	8	Integration Method	3	5	5	75
Sample Preparation	9	Operation Error	3	2	5	30
Acquisition	10	SW	2	5	3	30
Acquisition	11	O_1	2	5	3	30
Acquisition	12	D_8	1	5	5	25
Sample Preparation	13	Sample volume	2	2	5	20
Sample Preparation	14	NMR tube quality	3	1	5	15
Data Processing	15	Window Functions	3	1	5	15
Data Processing	16	Phase correction	5	2	1	10
Data Processing	17	Zero Filling	2	1	5	10
Instrument	18	Magnetic field uniformity	3	3	1	9
Instrument	19	Equipment stability	3	3	1	9
Data Processing	20	Baseline correction	3	2	1	6
Sample Preparation	21	Sample storage temperature	3	1	1	3
Sample Preparation	22	Sample storage time	3	1	1	3
Environmental	23	Ambient temperature	1	1	1	1
Environmental	24	Ambient Humidity	1	1	1	1
Environmental	25	Ambient air pressure	1	1	1	1
Environmental	26	Other Environmental factors	1	1	1	1
